# Lead Exposure in Free-Flying Turkey Vultures Is Associated with Big Game Hunting in California

**DOI:** 10.1371/journal.pone.0015350

**Published:** 2011-04-06

**Authors:** Terra R. Kelly, Christine K. Johnson

**Affiliations:** School of Veterinary Medicine, Wildlife Health Center, University of California Davis, Davis, California, United States of America; University of Lethbridge, Canada

## Abstract

Predatory and scavenging birds are at risk of lead exposure when they feed on animals injured or killed by lead ammunition. While lead ammunition has been banned from waterfowl hunting in North America for almost two decades, lead ammunition is still widely used for hunting big game and small game animals. In this study, we evaluated the association between big game hunting and blood lead concentration in an avian scavenger species that feeds regularly on large mammals in California. We compared blood lead concentration in turkey vultures within and outside of the deer hunting season, and in areas with varying wild pig hunting intensity. Lead exposure in turkey vultures was significantly higher during the deer hunting season compared to the off-season, and blood lead concentration was positively correlated with increasing wild pig hunting intensity. Our results link lead exposure in turkey vultures to deer and wild pig hunting activity at these study sites, and we provide evidence that spent lead ammunition in carrion poses a significant risk of lead exposure to scavengers.

## Introduction

Lead poisoning was recognized as an important cause of mortality in wildlife in the late 1950s [1]–[3] when ingested spent lead ammunition and fishing sinkers were linked to significant die-offs in waterfowl [4]. In 1986, a phase-in of nontoxic ammunition was initiated for waterfowl hunting in wetlands along the most impacted flyways in North America, followed by a nationwide ban of lead-based ammunition for waterfowl hunting in 1991 [5]. A major impetus for this policy change was mortality in the endangered bald eagle (*Haliaeetus leucocephalus*) population due to secondary lead poisoning from feeding on waterfowl containing lead ammunition [6]–[8]. While this regulation had a major impact on decreasing lead-associated mortality in waterfowl [9], there was no change in the prevalence of lead poisoning in eagles admitted to a rehabilitation center in the Midwestern U.S. during the post-ban period from 1991–1995. The authors attributed the lead poisoning in these cases in part to lead ammunition used in deer hunting [8].

Studies have demonstrated that lead bullets, upon impact, can produce hundreds of small fragments contaminating animal carcasses and discarded viscera that serve as important food sources for scavengers [10]–[13]. Predatory and scavenging birds are at risk of lead exposure when they consume embedded lead shot or bullet fragments in carcasses and gut-piles left in the field by hunters [6], [8], [14]–[16]. Old and new world vultures may be at increased risk of lead exposure because of their unique feeding ecology as obligate scavengers.

Lead-related mortality was a factor in the decline of the endangered California condor (*Gymnogyps californianus*) population in the 1980s [17] and has undermined efforts to establish a naturally sustainable population in the wild. California condors are monitored for lead exposure and a high proportion of the population exhibits elevated blood lead concentration during routine screening [18], [19], with some individuals requiring clinical intervention. The major challenges facing the recovery of this species have brought significant awareness to the policies surrounding the use of lead ammunition for hunting in North America, and in 2008 led to a ban of lead ammunition for most hunting activities within the California range of the condor. This legislation has received scrutiny from opponents who question whether lead exposure in wildlife is related to hunting activities and from wildlife agencies considering similar regulations to protect other vulnerable species.

Increased lead exposure during the deer hunting season has been detected in condors [18]–[20] and golden eagles sampled in the California range of the condor [21], however elevated blood lead concentrations have also been noted outside of the deer hunting season [21]. In Arizona and Utah, this seasonal increase in blood concentration in condors coincides with movement of the population to an area with high deer hunting pressure [20]. Temporal spatial correlations between big game hunting and lead exposure have also been documented in common ravens (*Corvus corax*) in the Greater Yellowstone Area. The ravens were observed to have significantly higher blood lead concentrations during the big game hunting season compared to the off season and lead exposure was found to be temporally correlated with big game hunting pressure [22]. In this study, food resources during the hunting season were limited by harsh weather and landscape, and hunter-killed prey was thought to be the primary food source available to ravens.

In California, year-round food sources for avian scavengers appear to be highly diverse due to a wide range of natural habitats, a mild climate, and a productive livestock economy. Turkey vultures (*Cathartes aura*) in California feed on a wide array of carrion including birds, reptiles, and small mammals, as well as large mammals, such as domestic livestock, wild ungulates, and beachcast marine animals [23]. Big game hunting in California is presumed to supply a substantial food source to avian scavengers, especially year-round wild pig hunting, which provides hunter-killed carrion throughout the year to scavengers within the wild pig range. Hunting activities vary by type and intensity throughout California and there is considerable overlap of different hunting seasons.

While previous studies have explored temporal and spatial patterns of lead exposure in avian scavengers with respect to confined hunting activities under conditions with limited food resources, data is currently lacking on lead exposure in avian scavengers in diverse habitats with varying big game hunting activity and a range of available food resources. Furthermore, blood lead concentrations have not been evaluated in association with hunting activities in free-flying populations of turkey vultures, an important scavenger in the Americas with excellent potential as a sentinel species for monitoring the availability of lead ammunition in carrion. In this study we tested the hypotheses that: (1) turkey vulture blood lead concentrations are higher during deer hunting season in an area with intense deer hunting compared to outside of the deer hunting season at the same location, and (2) turkey vulture blood lead concentrations are positively correlated with wild pig hunting intensity. Our results indicate that lead exposure in turkey vultures is highly associated with big game hunting activity despite a naturally diverse field setting and wide range of available food sources.

## Materials and Methods

### Ethics statement

Animal capture and sampling protocols were covered under state and federal permits (United States Geological Survey federal bird banding permit # 20431 and California Department of Fish and Game scientific collecting permit # 000221) and approved by the University of California, Davis Institutional Animal Care and Use Committee (protocol # 07-12955).

### Study site selection

Deer and wild pig hunting constitute the majority of the total statewide harvest of big game animals in California. Deer hunting occurs in late summer and fall in discrete seasons that vary by region. Pig hunting occurs year-round, although October through May tends to be the most popular time for hunting since pigs are most easily tracked in the wet season [24]. We evaluated the distribution of deer and wild pig hunting activity in California by overlaying county level harvest data from the 2006 game take hunter survey [25] and hunting seasons reported in the California mammal hunting regulations booklet [26]. The game take hunter survey is conducted annually by the California Department of Fish and Game and provides the best game harvest estimates for California [25]. We calculated the proportion of total statewide harvest for deer and wild pig hunting for each county as a relative measure of local hunting intensity.

We selected our study sites to represent a range of hunting activity in areas with a resident turkey vulture population. Turkey vultures were sampled during discrete periods outside of the reported turkey vulture migration [23] to ensure blood lead concentration reflected local lead exposure. We assumed that turkey vultures were feeding on hunter-killed carcasses and gut-piles within an area roughly equivalent to their estimated home range in each direction from our study sites [27]. We selected a site at the University of California Hopland Research Extension Center (HREC) in Mendocino County, California (38°59′39″N, 123°04′02″W) to evaluate the association between deer hunting and blood lead concentration in turkey vultures. This is a rural farming area situated amongst oak covered coastal foothills and is characterized by high intensity deer hunting, with Mendocino County accounting for the highest proportion of the deer harvest in California in 2006. We sampled turkey vultures at this site on two occasions; 1) a two week period beginning one week following the opening day of the deer hunting season, and 2) a two week period one month preceding the deer hunting season during the following year. The only hunting activity that varied between sampling efforts at this site was deer hunting. The black bear hunting season occurs concurrently with the deer hunting season in California. However, this site is not considered to be suitable habitat for black bears and there were no reports of bears harvested within our study area. Year-round hunting activities, including wild pig, rabbits, and non-game (coyotes, ground squirrels, skunks, opossum, starlings), were occurring during both sampling periods. The seasons for upland game and waterfowl hunting occur later in the fall and did not overlap with our sampling activities at this site.

**Figure 1:**
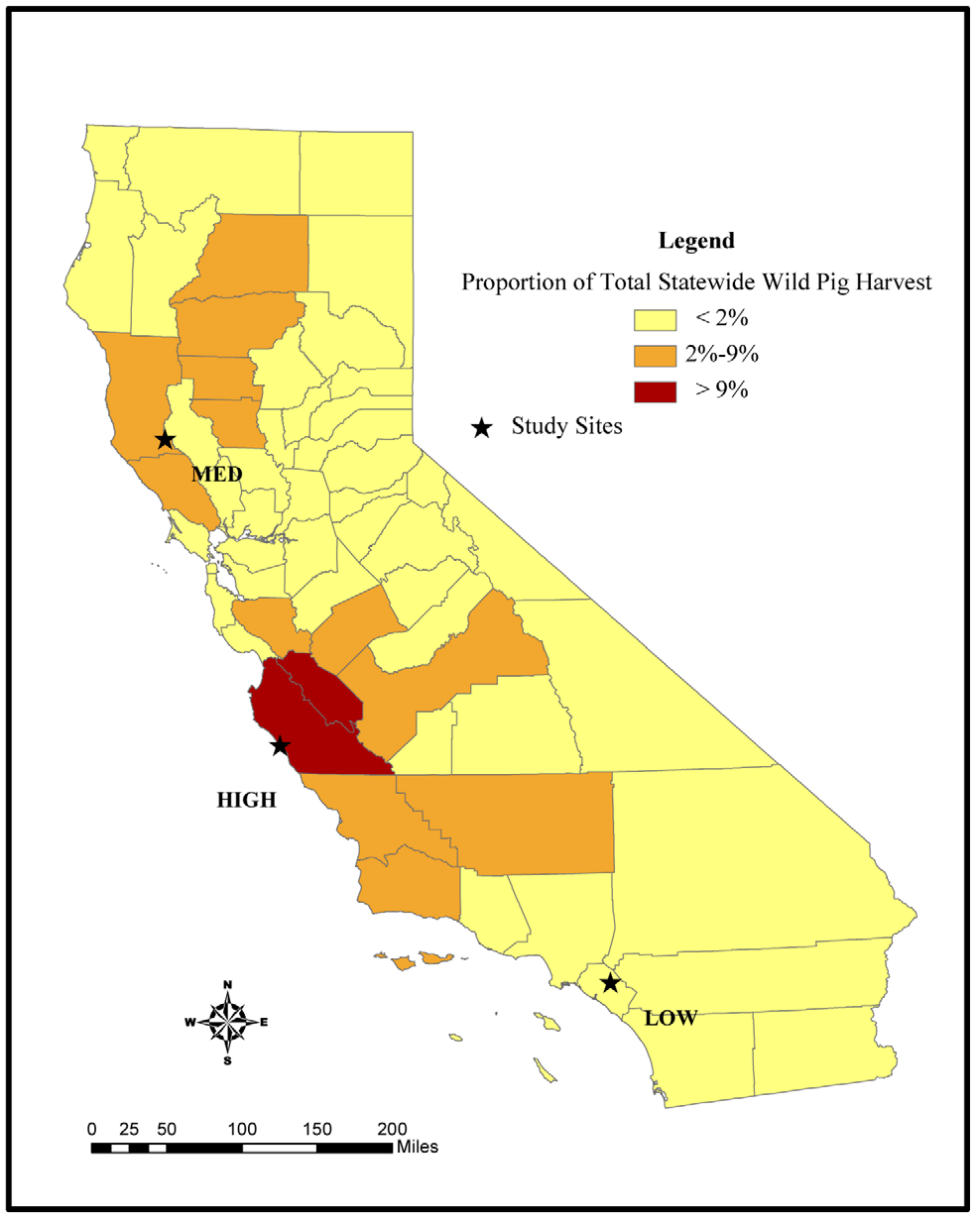
Map of study sites selected along a gradient of wild pig hunting intensity. The counties are shaded according to categories of the proportion of total statewide harvest of wild pigs by county.

We also selected three study sites along a gradient of wild pig hunting intensity in areas without other substantial hunting activities occurring at the time of sampling. Our low wild pig hunting intensity (LOW PIG) study site was located near Irvine Lake, an urban area in Orange County, California (33°45′44″N, 117°42′47″W) (Fig. 1). This area accounted for <1% of the total statewide harvest for wild pig hunting. Our medium pig hunting intensity (MED PIG) study site was located in Mendocino County at the same site that we used to evaluate the association between lead exposure and deer hunting (Fig. 1). This site accounted for approximately 3% of the total statewide harvest for wild pig hunting. Sampling occurred outside of the deer hunting season at this site in order to assess lead exposure in vultures due to medium intensity pig hunting. Lastly, our high intensity (HI PIG) study site was located at the University of California Landels-Hill Big Creek Reserve in Monterey County (36°03′51″N, 121°34′28″W) (Fig. 1), an area characterized by rugged oak-covered coastal mountains near the Big Sur coast surrounded by private and public land with high intensity wild pig hunting. Monterey County had the highest wild pig hunting pressure in 2006, accounting for greater than 15% of the total statewide reported pig harvest.

We focused our investigations on deer and wild pig hunting activities by sampling outside of other big game, upland game, and small mammal game hunting seasons. Year round non-game hunting activities were occurring within all of our study areas, however harvest rates were considerably lower than that for game species with managed hunting seasons during confined time periods. Furthermore, harvest data for non-game species was similar among sites [25].

### Sample collection and analysis

We captured turkey vultures in 2008 and 2009 using carrion baited walk-in traps with a “lure” turkey vulture, drop-in traps, and a ground net launcher (CODA Enterprises, Mesa, AZ). All vultures underwent basic health screening at the time of capture. Data collected on each vulture included capture location (GIS coordinates), sex, age class, body weight, basic morphometric measurements, and body condition score. The body condition score was a subjective measure of body condition index based on the contour of the pectoral muscle mass ranging from a score of 1 (emaciated) to 5 (obese). We determined the age class of each vulture as hatch year (HY), second year (SY), and after second year (ASY) by coloration of the head and maxilla [28]. Turkey vultures were marked with vinyl patagial tags or passive integrated transponders (AVID microchip system®, Avid Identification Systems, Inc., CA) in order to identify specific individuals at our study sites. We identified the sex of each vulture by polymerase chain reaction analysis of chromosomal DNA in blood samples (Sex Made Easy™, Zoogen Incorporated, Davis, CA).

Blood lead concentration rises within days of exposure to lead [29], thus serving as a relative indicator of the amount of lead available in carrion food sources at each study site. We considered blood lead concentrations ≤10 µg/dL as consistent with exposure to environmental background sources of lead, and concentrations >10 µg/dL indicating elevated exposure to a point source of lead, as would occur with ingestion of lead fragments. This threshold value was chosen based on blood lead concentrations <2 µg/dL in control turkey vultures and <10 µg/dL in control bald eagles in experimental lead dosing studies [29], [30], and lead concentrations of <4 µg/dL in pre-release California condors in captivity [31]. Furthermore, free-flying common ravens sampled outside of the big game hunting season had a median blood lead concentration of1.8 µg/dL [22]. Blood was collected from the brachial vein into lithium heparin blood tubes (Becton Dickinson, Franklin Lakes, NJ) and stored at −80°C until analysis. Blood samples were analyzed for lead concentration at the California Animal Health and Food Safety Laboratory (CAHFS), University of California, Davis using graphite atomic absorption spectrophotometry (PerkinElmer Model AAnalyst 800 graphite furnace atomic absorption spectrophotometer; PerkinElmer, Waltham, MA). To ensure precision of results, all samples were run in duplicate and results were considered acceptable when the relative standard deviation was ≤10%. To ensure accuracy of results, a proficiency testing blood sample from the Wisconsin State Laboratory of Hygiene with a target lead value was analyzed with each set of blood samples. These WSLH samples were within one standard deviation of the target value. The lower laboratory reporting limit for blood lead concentration was 6 µg/dL.

### Data analysis

Blood lead concentration data were left censored since concentrations falling below the laboratory limit of 6 µg/dL were reported as a “nondetect” (<6 µg/dL). Data were therefore analyzed using NADA (Nondetects And Data Analysis) [32], a library package in R [33] that provides an analytical framework for analyzing left-censored data. This framework allows for censored data to be incorporated into computations of the statistics using nonparametric and parametric methods. The probability distribution of the blood lead concentration data was assessed using probability plots and the Shapiro-Wilks test. A significance level of 0.05 was used for all statistical analyses unless stated otherwise.

Because age and sex related differences in blood lead concentrations have been reported in other studies [34]–[36], we evaluated differences in median turkey vulture blood lead concentration by sex using the Wilcoxon rank sum test, and age class using the Kruskal-Wallis test with post hoc Mann- Whitney U test comparisons, including correction for multiple comparisons of the respective p-values [37].

We used linear regression models with maximum likelihood estimation, that incorporate censored data points, to investigate the relationship between vulture blood lead concentration and deer hunting and wild pig hunting intensity, separately. To identify the most parsimonious models, we used the likelihood-ratio test to determine whether each variable and interaction term significantly improved model fit (P<0.05), compared to a model without that variable. Variables and interaction terms were retained in the model if they improved model fit, while minimizing Akaike's information criterion (AIC), or were determined to be important confounders based on a change in parameter estimates from the crude parameter by at least 10% with addition of the variables to the model. Overall model fit was assessed by evaluation of the residual plots.

To further illustrate the association between vulture blood lead exposure and hunting activity, we calculated the overall prevalence of elevated blood lead exposure (≥10 µg/dL), and the prevalence for each study group of interest with estimates of 95% confidence intervals using binomial probability testing. We also demonstrated a trend in blood lead concentration across levels of wild pig hunting intensity using a nonparametric test for trend [38].

## Results

A total of 172 turkey vultures were captured for this study, including 90 ASY females, 67 ASY males, 4 SY males, 8 HY females, and 3 HY males. Age classifications were collapsed into two categories, HY vultures and after hatch year (AHY), vultures given that median blood lead concentrations did not differ between second year vultures and adults. Overall, 48% (83/172, 95% CI = 41%–55%) of our turkey vultures had elevated blood lead concentrations >10 µg/dL. Despite blood lead concentrations consistent with acute lead toxicosis (≥100 µg/dL) [39] in some individuals, none of the vultures were showing signs of intoxication at capture.

### Effect of Deer Hunting Season on Blood Lead Concentration

**Figure 2:**
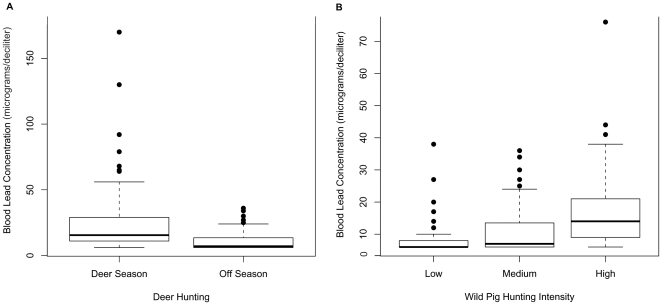
Distribution of blood lead concentrations (µg/dL) in turkey vultures across study sites with varying big game hunting activities. The rectangle represents the interquartile range (IQR) from the first quartile (the 25th percentile) to the third quartile (the 75th percentile). The whiskers extend out to the smallest value within 1.5 times the IQR from the first quartile and the largest value within 1.5 times the IQR from the third quartile. The dots represent outlying data points. **A**. Turkey vultures sampled during the deer hunting season had significantly higher blood lead concentration than vultures sampled outside of the deer hunting season (*P*<0.01). **B**. There was a significant positive trend in turkey vulture blood lead concentrations across wild pig hunting intensity categories (*P*<0.0001).

We sampled 34 vultures during the deer hunting season and 39 vultures outside of the deer hunting season at our site with high intensity deer hunting. The median blood lead concentration was 15 µg/dL (range: 6–170 µg/dL) during deer hunting season compared to 7 µg/dL (range: 6–36 µg/dL) outside of the deer hunting season (Figure 2A). There was a significant relationship between deer hunting and turkey vulture blood lead concentration in our multivariable analysis (Likelihood Ratio Chi-Square test, G = 20.64, d.f. = 3, *P* = 0.0001, Table 1A) and significantly higher blood lead concentrations (3-fold difference in the geometric mean blood lead concentration) during the deer hunting season compared to outside of the deer hunting season (*P*<0.01). An interaction term between deer hunting season and age class was fit in the model to account for the difference in the effect of deer hunting on blood lead concentration by age. Specifically, only after hatch year vultures had higher blood lead concentrations during the deer hunting season. We found no difference in blood lead concentration between sexes among vultures captured at this location. The prevalence of elevated blood lead exposure in vultures sampled during the deer hunting season was 76% (26/34, 95% CI = 60%–88%), which was significantly higher than the 36% prevalence among vultures sampled outside of the deer hunting season (14/39, 95% CI = 22%–51%). Twenty-one percent of vultures (7/34, 95% CI = 9%–36%) sampled during the deer hunting season had moderate levels of elevated blood lead exposure (≥60 µg/dL), and two of the vultures had blood lead concentrations >100 µg/dL, which are levels consistent with acute lead intoxication [39]. None of the vultures sampled outside of the deer hunting season had blood lead concentrations ≥60µg/dL.

**Table 1:** Regression estimates for the effect of big game hunting activity on turkey vulture blood lead concentrations (µg/dL).

A. Deer Hunting Season Model:	Parameter estimate*	Standard error	P-value
Intercept	2.01	0.16	<0.001
Deer hunting season	1.06	0.23	<0.001
Age class (hatch year)	0.82	0.92	0.300
Deer hunting season * age class (hatch year)	−2.03	1.05	0.050
**B. Wild Pig Hunting Intensity Model:**			
Intercept	1.49	0.15	<0.001
Wild pig hunting (medium intensity)	0.45	0.19	0.010
Wild pig hunting (high intensity)	1	0.18	<0.001
Sex (males)	0.26	0.14	0.040
Age class (hatch year)	−0.61	0.45	0.100

*Presented on natural logarithmic scale.

### Effect of Wild Pig Hunting Intensity on Blood Lead Concentration

We sampled 52 vultures at our LOW PIG site, 39 vultures at our MED PIG site and 47 at our HI PIG site. The median blood lead level was 4 µg/dL (range: 6–38 µg/dL) at the LOW PIG site, 7 µg/dL (range: 6–36 µg/dL) at the MED PIG site, and 14 µg/dL (range: 6–76 µg/dL) at the HI PIG site (Figure 2B). We detected a significant relationship between wild pig hunting intensity and turkey vulture blood lead concentration in our age and sex adjusted model (Likelihood Ratio Chi-Square test, G = 42.81, d.f. = 4, *P*<0.0001, Table 1B). The geometric mean for blood lead concentration (µg/dL) was higher at our MED PIG and HI PIG sites compared to our LOW PIG site, by a factor of 2 and 3, respectively. Age class and sex were incorporated in the model to adjust for joint confounding. Males at these three sites had significantly higher blood lead concentration compared to females (*P* = 0.04). We also detected a positive trend in vulture lead exposure across wild pig hunting intensity categories using the non-parametric test for trend (z = 6.78, *P*<0.0001). The prevalence of elevated blood lead exposure (>10 µg/dL) was 13% (7/52, 95% CI = 6%–24%) in vultures sampled at the LOW PIG site, 36% (14/39, 95% CI = 22%–52%) in vultures sampled at the MED PIG site, and 66% (31/47, 95% CI = 53%–78%) in vultures sampled at the HI PIG site.

## Discussion

Our findings provide evidence that big game hunting in California increases the risk of lead exposure in avian scavengers from ingestion of lead ammunition. Elevated blood lead concentration in free-flying turkey vultures varied according to deer and wild pig hunting activities occurring at our study sites. Turkey vultures captured during the deer hunting season experienced significantly higher levels of lead exposure compared to vultures captured outside of the deer hunting season with concentrations consistent with intoxication in some individuals. None of the vultures sampled outside of the deer hunting season at the same location had similarly high levels. Turkey vulture blood lead concentrations were also highly correlated with wild pig hunting intensity with concentrations three times higher at our high intensity wild pig hunting site compared to our site with low wild pig hunting intensity. Capture locations used in our study were carefully selected such that deer and wild pig hunting activities were the major factors varying between sampling efforts. We sampled outside of other big game, upland game, and small mammal game hunting seasons and the total year-round harvest reported for non-game species was similar among sites.

The elevated blood lead concentrations documented in turkey vultures captured for our study is consistent with exposure to a point source of concentrated lead during foraging. Unlike some other contaminants, lead does not undergo biological magnification in the food chain, or concentrate in animal blood or tissues at levels high enough to cause significant secondary lead poisoning in predators or scavengers. Lead concentration can achieve high levels in the environment (water and soil) in small localized areas around lead mines, smelters and industrial plants [40]–[44]. However, lead poisoning in wildlife from these concentrated environmental sources is relatively rare in occurrence. Furthermore, there were no lead-related industrial activities in the vicinity of our field sites, so these sources were not likely to have contributed to lead exposure in the vultures captured for this study. Instead, the patterns in lead exposure observed in our study attribute the point source of elevated blood lead concentrations to lead fragments in hunted animal carcasses available to foraging vultures during big game hunting activities.

During our field efforts, turkey vultures were observed feeding on wild mammals and domestic animal carrion. Vultures at all study sites had access to dead domestic animals from nearby livestock operations; our LOW PIG and MED PIG sites were near major cattle and sheep operations [45] and our HI PIG site was in close proximity to designated California condor feeding stations where dairy calf carcasses are put out as a source of food for condors. All sites were also within flight distance to the coast where beachcast marine animals are available to scavengers. Marine mammals occasionally die with gunshot wounds [46], thus serving as potential sources of lead exposure to scavengers. We assume that the same is true for livestock in California; however we were unable to find reports of gunshot wounds as a cause of mortality in these animals [47]–[49]. Most likely, these occurrences are comparatively infrequent and would only vary systematically across our study locations along the gradient of big game hunting intensity if accidental shooting of livestock increases during big game hunting activities.

This study is the first to document blood lead concentrations in wild populations of turkey vultures and measure an association between lead exposure in turkey vultures and hunting activities. As obligate scavengers, turkey vultures may be at higher risk of lead exposure from ingestion of spent ammunition, compared to predators and scavengers that do not rely on carrion as a sole food source. Our findings suggest that turkey vultures rely heavily on hunted wild animal carrion for food. Interestingly, deleterious effects associated with elevated lead exposure were not observed in captured turkey vultures, despite observed blood lead concentrations in some individuals that have been reported to cause lead toxicity and death [29]. However, intensive long term follow-up of individual birds with telemetry and frequent recaptures would be necessary to detect clinical signs associated with lead intoxication and lead-associated decreases in survivorship. Experimental intoxication of turkey vultures has shown that this species can tolerate a substantial burden of lead exposure and must ingest more lead to reach high levels in the blood and cause mortality than levels that have been reported in other avian species [29]. While turkey vultures may be a poor physiological model for the toxic effects of lead [29], they are good sentinels for monitoring lead exposure from ingestion of lead ammunition in field settings due to their feeding ecology and potentially high survival rates despite high levels of lead exposure.

In studies of raptors and vultures collected in Canada, turkey vultures had the highest tissue levels of lead compared to other species [50], [51]. Turkey vultures have been found to also have significantly elevated bone lead concentrations, consistent with chronic exposure [52]. Chronic lead exposure from prolonged or repeated exposure at lower concentrations can have sublethal effects in avian species by impairing reproductive success [53], growth rate of young birds [54], [55], neurobehavioral function [56], [57], immunity [58], [59], and physiology [29], [60]. Furthermore, a number of sublethally-exposed birds likely die from other causes in which lead could be a contributing factor. As a result of this myriad of deleterious health effects, birds may be more susceptible to other stressors and increased risk of predation and disease. While the population impacts of acute and chronic lead exposure are unknown for most wild bird populations, the effect of lead exposure on long-term sustainability through direct mortality and sublethal effects is a concern.

Ingestion of spent ammunition has been linked to lead exposure and lead poisoning in a range of wildlife species worldwide [16], [61]–[63]. This study contributes to the growing body of literature emphasizing the hazards that lead ammunition poses to both wildlife and public health [63]. Non-lead ammunition alternatives are increasingly available for both small and large game species. Transition to alternatives for shooting game and non-game species will benefit many wildlife species and will also eliminate the potential risk to humans of accidental ingestion of lead in harvested game [64].

## References

[1] Bellrose F (1959). Lead poisoning as a mortality factor in waterfowl
populations.. Illinois Natural History Survey Bulletin.

[2] Irwin J, Karstad L (1972). The toxicity for ducks of disintegrated lead shot in a
simulated-marsh environment.. Journal of Wildlife Diseases.

[3] Sanderson G, Bellrose F (1986). A review of the problem of lead poisoning in
waterfowl.. Illinois Natural History Survey, Champaign, Illinois.

[4] Bates F, Barnes D, Higbee J (1968). Lead toxicosis in mallard ducks.. Bulletin of the Wildlife Disease Association.

[5] United States Fish and Wildlife Service (2010). Nontoxic shot regulations for hunting waterfowl and coots in the
U.S.. http://www.fws.gov/migratorybirds/currentbirdissues/nontoxic.htm.

[6] United States Fish and Wildlife Service (1986). Use of lead shot for hunting migratory birds in the United
States: Final Supplemental Environmental Impact Statement..

[7] Kendall R, Lacher T, Bunck C, Daniel B, Driver C (1996). An ecological risk assessment of lead shot exposure in
non-waterfowl avian species: upland game birds and raptors.. Environmental Toxicology and Chemistry/SETAC.

[8] Kramer J, Redig P (1997). Sixteen years of lead poisoning in eagles, 1980–95: An
epizootiologic view.. Journal of Raptor Research.

[9] Anderson W, Havera S, Zercher B (2000). Ingestion of lead and nontoxic shotgun pellets by ducks in the
Mississippi flyway.. The Journal of Wildlife Management.

[10] Hunt W, Burnham W, Parish C, Burnham K, Mutch B (2006). Bullet fragments in deer remains: implications for lead exposure
in avian scavengers.. Wildlife Society Bulletin.

[11] Knopper L, Mineau P, Scheuhammer A, Bond D, McKinnon D (2006). Carcasses of shot Richardson's ground squirrels may pose
lead hazards to scavenging hawks.. The Journal of Wildlife Management.

[12] Pauli JN, Buskirk SW (2007). Recreational Shooting of Prairie Dogs: A portal for lead entering
wildlife food chains.. Journal of Wildlife Management.

[13] Stephens R, Johnson A, Plumb R, Dickerson K, McKinstry M, Watson R, Fuller M, Pokras M, Hunt W (2009). Risk assessment of lead poisoning in raptors caused by
recreational shooting of prairie dogs.. Ingestion of Lead from Spent Ammunition: Implications for Wildlife and
Humans.

[14] Gill C, Langelier K (1994). Acute lead poisoning in a bald eagle secondary to bullet
ingestion.. Canadian Veterinary Journal.

[15] Locke L, Thomas N, Fairbrother A, Locke L, Hoff G (1996). Lead poisoning of waterfowl and raptors..

[16] Pain D, Fisher I, Thomas V, Watson R, Fuller M, Pokras M, Hunt W (2009). A global update of lead poisoning in terrestrial birds from
ammunition sources.. Ingestion of Lead from Spent Ammunition: Implications for Wildlife and
Humans.

[17] Snyder N, Snyder H (2000). The California condor: a saga of natural history and
conservation.

[18] Hall M, Grantham J, Posey R, Mee A, Mee A, Hall L (2007). Lead exposure among reintroduced California condors in southern
California.. California condors in the 21st century. Series in Ornithology no.
2.

[19] Sorenson K, Burnett J, Mee A, Hall L (2007). Lead concentrations in the blood of Big Sur California
condors.. California condors in the 21st century.

[20] Hunt W, Parish C, Farry S, Lord T, Sieg R, Mee A, Hall L (2007). Movements of introduced California condors in Arizona in relation
to lead exposure.. California condors in the 21st century. Series in Ornithology no.
2.

[21] Pattee O, Bloom P, Scott J, Smith M (1990). Lead hazards within the range of the California
Condor.. The Condor.

[22] Craighead D, Bedrosian B, Watson RT, Fuller M, Pokras M, Hunt W (2009). A relationship between blood lead levels of common ravens and the
hunting season in the southern Yellowstone ecosystem.. Ingestion of Lead from Spent Ammunition: Implications for Wildlife and
Humans.

[23] Kirk DA, Mossman M, Poole A, Gill F (1998). Turkey vulture (*Cathartes aura*).. The Birds of North America, No. 339.

[24] Waithman J (2001). Guide to hunting wild pigs in California: 44.. http://www.dfg.ca.gov/publications/docs/pigguide.pdf.

[25] California Department of Fish and Game (2006). Report of the 2006 game take hunter survey.. http://www.dfg.ca.gov/wildlife/hunting/uplandgame/reports/docs/surveys/2000-2009/2006HS.pdf.

[26] California Department of Fish and Game (2007). Hunting and Sport Fishing Regulations.. http://www.dfg.ca.gov/regulations/.

[27] Coleman J, Fraser J (1989). Habitat use and home ranges of black and turkey
vultures.. The Journal of Wildlife Management.

[28] Henckel E (1981). Ageing the turkey vulture.. North American Bird Bander.

[29] Carpenter J, Pattee O, Fritts S, Rattner B, Wiemeyer S (2003). Experimental lead poisoning in turkey vultures (*Cathartes
aura*).. Journal of Wildlife Diseases.

[30] Hoffman DJ, Pattee OH, Wiemeyer SN, Mulhern B (1981). Effects of lead shot ingestion on d-aminolevulinic acid
dehydratase activity, hemoglobin concentration and serum chemistry in bald
eagles.. Journal of Wildlife Diseases.

[31] Church ME, Gwiazda R, Risebrough RW, Sorenson K, Chamberlain CP (2006). Ammunition is the principal source of lead accumulated by
California Condors re-introduced to the wild.. Environmental Science and Technology.

[32] Lopaka L (2009). NADA: Nondetects And Data Analysis for environmental
data.. http://cran.r-project.org/package=NADA.

[33] R Development Core Team (2009). R: A language and environment for statistical
computing.. http://www.R-project.org.

[34] Pain D, Amiard-Triquet C, Bavoux C, Burneleau G, Eon L (1993). Lead poisoning in wild populations of marsh harriers
*Circus aeruginosus* in the Camargue and
Charente-Maritime, France.. Ibis.

[35] Wayland M, Bollinger T (1999). Lead exposure and poisoning in bald eagles and golden eagles in
the Canadian Prairie Provinces.. Environmental Pollution.

[36] Craighead D, Bedrosian B (2008). Blood lead levels of common ravens with access to big-game
offal.. Journal of Wildlife Management.

[37] Holm S (1979). A simple sequentially rejective multiple test
procedure.. Scandinavian Journal of Statistics.

[38] Cuzick J (1985). A Wilcoxon-type test for trend.. Statistics in Medicine.

[39] Franson J, Beyer W, Heinz G (1996). Interpretation of tissue lead residues in birds other than
waterfowl.. Redmon-Norwood A. Environmental Contaminants in Wildlife.

[40] Henny C, Blus L, Hoffman D, Grove R, Hatfield J (1991). Lead accumulation and osprey production near a mining site on the
Coeur d'Alene River, Idaho.. Archives of Environmental Contamination and Toxicology.

[41] Blus L, Henny C, Hoffman D, Grove R (1991). Lead toxicosis in tundra swans near a mining and smelting complex
in northern Idaho.. Archives of Environmental Contamination and Toxicology.

[42] Henny CJ, Blus LJ, Hoffman DJ, Grove RA (1994). Lead in hawks, falcons and owls downstream from a mining site on
the Coeur d'Alene River, Idaho.. Environmental Monitoring and Assessment.

[43] Blus LJ, Henny CJ, Hoffman DJ, Audet DJ (1999). Persistence of high lead concentrations and associated effects in
tundra swans captured near a mining and smelting complex in northern
Idaho.. Ecotoxicology.

[44] Sileo L, Creekmore LH, Audet DJ, Snyder MR, Meteyer CU (2001). Lead poisoning of waterfowl by contaminated sediment in the Coeur
d'Alene River.. Archives of Environmental Contamination and Toxicology.

[45] Pineda-krch M, Thunes C, Carpenter TE (2010). Potential impact of introduction of foot-and- mouth disease from
wild pigs into commercial livestock premises in California.. American Journal of Veterinary Research.

[46] Greig DJ, Gulland FMD, Kreuder C (2005). A decade of live California sea lion (*Zalophus
californianus*) strandings along the central California coast:
causes and trends, 1991–2000.. Aquatic Mammals.

[47] United States Department of Agriculture (2005). Sheep and lamb death loss in the United States,
1999..

[48] United States Department of Agriculture (2010). Mortality of calves and cattle on U.S. beef cow-calf
operations..

[49] Gardner IA, Hird DW, Utterback WW, Danaye-Elmi C, Heron BR (1990). Mortality, morbidity, case-fatality, and culling rates for
California dairy cattle as evaluated by the National Animal Health
Monitoring System, 1986–1987.. Preventative Veterinary Medicine.

[50] Martin P, Campbell D, Hughes K, McDaniel T (2008). Lead in the tissues of terrestrial raptors in southern Ontario,
Canada, 1995–2001.. Science of the Total Environment.

[51] Clark AJ, Scheuhammer A (2003). Lead poisoning in upland-foraging birds of prey in
Canada.. Ecotoxicology.

[52] Wiemeyer S, Jurek R, Moore J (1986). Environmental contaminants in surrogates, foods, and feathers of
California condors (*Gymnogyps
californianus*).. Environmental Monitoring and Assessment.

[53] Buerger T, Mirarchi R, Lisano M (1986). Effects of lead shot ingestion on captive mourning dove
survivability and reproduction.. The Journal of Wildlife Management.

[54] Custer T, Franson J, Pattee O (1984). Tissue lead distribution and hematologic effects in American
kestrels (*Falco sparverius*) fed biologically incorporated
lead.. Journal of Wildlife Diseases.

[55] Hoffman D, Franson J, Pattee O, Bunck C, Anderson A (1985). Survival, growth, and accumulation of ingested lead in nestling
American kestrels (*Falco sparverius*).. Archives of Environmental Contamination and Toxicology.

[56] Burger J, Gochfeld M (2005). Effects of lead on learning in herring gulls: an avian wildlife
model for neurobehavioral deficits.. Neurotoxicology.

[57] Kelly A, Kelly S (2005). Are mute swans with elevated blood lead levels more likely to
collide with overhead power lines?. Waterbirds.

[58] Redig PT, Lawler EM, Schwartz S, Dunnette JL, Stephenson B (1991). Effects of chronic exposure to sublethal concentrations of lead
acetate on heme synthesis and immune function in red-tailed
hawks.. Archives of Environmental Contamination and Toxicology.

[59] Snoeijs T, Dauwe T, Pinxten R, Vandesande F, Eens M (2004). Heavy metal exposure affects the humoral immune response in a
free-living small songbird, the great tit (*Parus
major*).. Archives of Environmental Contamination and Toxicology.

[60] Gangoso L, Alvarez-Lloret P, Rodriguez-Navarro A, Mateo R, Hiraldo F (2009). Long-term effects of lead-poisoning on bone mineralization in
vultures exposed to ammunition sources.. Environmental Pollution.

[61] Pain D, Beyer W, Heinz G, Redman-Norwood A (1996). Lead in waterfowl.. Environmental Contaminants in Wildlife: Interpreting Tissue
Concentrations.

[62] Rogers T, Bedrosian B, Craighead D, Quigley H, Foresman K, Watson R, Fuller M, Pokras M, Hunt W (2009). Lead ingestion by scavenging mammalian carnivores in the
Yellowstone ecosystem.. Ingestion of Lead from Spent Ammunition: Implications for Wildlife and
Humans.

[63] Tranel MA, Kimmel RO, Watson R, Fuller M, Pokras M, Hunt W (2009). Impacts of lead ammunition on wildlife, the environment, and
human health-a literature review and implications for
Minnesota.. Ingestion of Lead from Spent Ammunition: Implications for Wildlife and
Humans.

[64] California Department of Fish and Game (2010). Certified non-lead ammunition.. http://www.dfg.ca.gov/wildlife/hunting/condor/certifiedammo.html.

